# The Effect of Ultrasound-Measured Preinduction Cervical Length on Delivery Outcome in a Low-Resource Setting

**DOI:** 10.1155/2020/8273154

**Published:** 2020-05-01

**Authors:** Chidebe C. Anikwe, Bartholomew C. Okorochukwu, Emmanuel Uchendu, Cyril C. Ikeoha

**Affiliations:** ^1^Department of Obstetrics & Gynaecology, Federal Teaching Hospital, Abakaliki, Ebonyi, Nigeria; ^2^Department of Obstetrics and Gynaecology, Federal Medical Centre, Owerri, Imo, Nigeria; ^3^Department of Radiology, Federal Teaching Hospital, Abakaliki, Ebonyi, Nigeria

## Abstract

**Background:**

Induction of labour is not without risk, and it calls for a method that will be sensitive enough to predict successful labour induction.

**Aim:**

This study aims to evaluate the role of transvaginal ultrasonographic cervical length measurement at term in the prediction of successful induction of labour (IOL).

**Materials and Methods:**

This prospective study was carried out in the Department of Obstetrics and Gynaecology of Federal Teaching Hospital Abakaliki between 1st of July and 30^th^ of November 2015. Preinduction Bishop score and cervical length were assessed before induction of labour. Intracervical, cervical, extraamniotic Foley catheter was used to improve the Bishop score. The data were analyzed using the IBM SPSS Statistics 20.

**Results:**

The mean maternal age of the study group was 30.68 ± 6.38 years with a range of 19–43 years. The mean gestational age and parity were 39.57 ± 1.49 and 1.85 ± 0.63, respectively. All the women studied had successful induction of labour with mean induction delivery time of 8.1 ± 3.0 hours and mean duration of labour of 7.4 ± 2.9 hours. Preinduction cervical length is a good predictor of a short duration of labour (*P* = 0.001). Parturient with a preinduction cervical length of less than 3 cm was likely to have labour lasting less than 6 hours (RR = 4.20 (95% CI 1.85–9.529).

**Conclusion:**

Transvaginal sonographic measurement of cervical length provides a useful prediction of the likelihood of duration of labour following the induction of labour. It is recommended that IOL should be considered and success anticipated in a parturient with a cervical length less than 3 cm.

## 1. Introduction

Labour and delivery are the focus and climax of the reproductive process, which could be achieved by artificial initiation of the labour process. Induction of labour (IOL) is indicated when the continuation of the pregnancy poses a great danger to the wellbeing of the mother, baby, or both [1]. The indications for labour induction should be specific and justifiable because of the associated increased risk of caesarean delivery [2, 3]. The incidence of induction of labour has increased worldwide and varies from region to region [4]. It is estimated that about 20% of pregnancies are induced for various medical reasons after weighing the risk and benefit of the induction of labour [5] with prolonged pregnancy being the commonest reason [6]. Labour induction is not without risk to both the mother and the fetus. The aim is to achieve vaginal delivery with the delivery of a healthy baby to a satisfied mother. This outcome is affected by the ripeness of the cervix as assessed by the Bishop score [6]. A favourable cervix reduces the >20% risk of caesarean delivery associated with induction of labour, especially in nulliparae [7]. It also reduces prolonged labour and failed induction. To date, the Bishop score (BS) has remained the standard method in the prediction of the duration and safety of induced labour. Its subjectiveness, poor reproducibility, interobserver and intraobserver difference, poor assessment of cervical length (CL) especially when the cervix is closed, and IOL outcome are some of its limitation [8, 9]. In addition, the supravaginal portion of the cervix, which makes about half of the cervix could not be assessed properly with the digital examination.

In an effort to predict successful induction of labour so as to avert caesarean delivery that might follow, transvaginal ultrasonographic measurement of cervical length was employed. It offers some advantages over the Bishop score in its ability to properly access the cervical length and initial changes at the internal os (even when the cervix is closed) and its shortening, which is a better representative of cervical effacement which is more accurate [10]. This is seen as the most important parameter to successful IOL after controlling for other confounders when using the Bishop score. Various studies on this subject have reported on the usefulness of ultrasound measured cervical length in predicting the mode of delivery. Tan et al. reported that both Bishop score and CL were significantly able to predict the need for caesarean section among the cohort of women they studied, but ultrasound measured CL prediction has a superior sensitivity but with a marginally better positive predictive value [11]. This effect was observed with CL of 0.2 cm or more and BS of 5 or less. This finding is in concordance with the reports by Nitesh Kanwar et al. in India [12], Gokturk et al. in Turkey [13], Hale Bahadori et al. in Iran [14], Verhoeven et al. in the Netherlands [8], and Pereira et al. in London [15]. Ben-Harush et al. [16] in a prospective study of 71 patients observed a statistically significant linear correlation between sonographic cervical lengths to IOL and labour duration. The cohort of women with CL of less than 28 mm significantly had a shorter induction delivery time compared to patients with more than 28 mm length. In a similar study although with a different route of CL measurement, Khazardoost et al. [17] also showed that higher cervical length is significantly associated with a parturient being delivered abdominally. On the other hand, there are studies with conflicting results on the above on the place of ultrasound measured CL versus BS in predicting IOL outcomes. The studies of Chandra et al. [18], Gonen et al. [19], Watson et al. [20], and Rozenberg et al. [21] showed no correlation between sonographic cervical length and induction delivery interval, duration of labour, successful induction, or length of the latent phase of labour.

To the best of our knowledge, there is a paucity of studies on the place of ultrasound-measured CL on the mode of delivery especially on women undergoing IOL in the study area. This could be attributed on the influence of the environment, the need and place of practice since ultrasound may not be readily available and affordable in resource-poor settings [22], thus encouraging research in areas of great need. The existence of this knowledge gap in the study area thus emphasizes the need for this research to help fill a knowledge gap on this subject. With increasing development and availability of ultrasound in Nigeria especially in a teaching hospital, the transvaginal ultrasonographic cervical measurement being quantitative, reproducible, and easy to learn might be employed in the evaluation of our obstetric population that would undergo IOL. This is important in triaging for induction of labour when the indication is “minor,” Bishop score not favourable, or there is an urgent need to induce labour as CL is thought to be a better representative of “ripeness of cervix” than Bishop score [23]. The study aims to evaluate the role of preinduction transvaginal cervical length measurement in predicting the induction delivery interval.

## 2. Materials and Methods

### 2.1. Study Background

The study was carried out in the Obstetrics and Gynaecology department of Federal Teaching Hospital, Abakaliki, Ebonyi State, Nigeria from 1^st^ July to 30^th^ November 2015. Federal Teaching Hospital Abakaliki is the only tertiary health institution in the state, receiving a referral from primary and secondary health care institutions in the state. The Department of Obstetrics and Gynaecology is managed by 27 consultants and 90 Resident doctors with the help of trained midwives/nurses. The study was approved by the Ethics and Research Committee of the hospital with ethical approval number FETHA/REC/VOL1/2014/123. Informed written consent was obtained from the women before inclusion in the study.

### 2.2. Study Participant

Ballot method of simple random sampling was used for the recruitment of the study population. The clients admitted for induction of labour was numbered, and the number placed in a nontransparent bag. A number was drawn from the bag with replacement and the client with the number selected for the study. Fifty (50%) percent of the clients admitted for IOL were selected for study each time, and in a situation where a client was admitted for IOL, she was selected for the study after consenting and having met the inclusion criteria.

### 2.3. Inclusion Criteria and Exclusion Criteria

The following pregnant women were included in the study: those that consented to participate, pregnant women with singleton fetus of normal weight and in cephalic presentation, induction of labour at term, a fetus with normal and reassuring fetal heart rate pattern, and parturient with no contraindication to vaginal delivery. Pregnant women excluded were parturient with contraindication to vaginal delivery, cases of antepartum haemorrhage, pregnant women with one or more previous caesarean section scar, HIV-positive pregnancy, and pregnant women with genital warts or herpes simplex infection.

Following the selection of a patient for the study, she was interviewed with a questionnaire. Some of the information obtained included the sociodemographic parameters, obstetrics parameters, and the indication for induction of labour. The neonatologist and theatre personnel were informed and consent obtained for the procedure. Vaginal examination was done to assess the favourability of Bishop score for induction of labour. Parturients with unfavourable Bishop score for induction of labour had their cervix ripened with intracervical extraamniotic Foley catheter.

### 2.4. Cervical Ripening

Aseptic procedure was used in the insertion of a Foleys' catheter for cervical ripening. This was inserted at 18:00 hours (6 pm) and removed at 06:00 hours (6 am) the following day. Removal of the catheter was done when it fell out of the cervix before 06:00 hours. Passage of extraamniotic intracervical Foleys' catheter was repeated to a maximum of three times. Digital vaginal examination was done to determine the baseline Bishop score. For the passage of extraamniotic intracervical Foleys' catheter, the cervix was exposed with a sterile Graves' or Cusco's speculum, inspected, and with the help of an assistant, Foleys' catheter was opened up and taken from the pack. It was held about 1.5 cm from the balloon point with a sponge-holding forceps and gently introduced with the tip pointing posteriorly to avoid rupturing the fetal membranes. It is gently advanced until all the balloon point and about 1–1.5 cm below was taken up. The catheter was inflated with 30 ml of sterile water. It was plastered to the nondependent thigh after a little torque was applied. It was spigotted.

### 2.5. Preinduction Cervical Measurement

A preinduction cervical length measurement was done using an ultrasound (Mindray DC—N6, 3/3.5 MHz probe transvaginal ultrasound scanner; Shenzhen Mindray China, 2012) by consultant radiologist. He was blinded to the digital cervical assessment of Bishop score. The clients were placed in the lithotomy position, covered with a gown, with an empty bladder (having being asked to urinate before the procedure) to avoid elongation of the cervix. The transvaginal probe, covered with a condom and sonographic gel applied, was placed about 3 cm away from the cervix to avoid distortion of the cervical position and shape. Cervical length was measured according to a standard technique described by Andersen [24].

### 2.6. Labour Management

Patients for induction of labour were admitted into the antenatal ward (for at least 12 hours) during which haemoglobin concentration was estimated, and two units of blood were cross-matched. A cervical assessment was done to determine the inducibility of the cervix and the need for cervical ripening. Induction of labour was commenced in the morning between 08:00-09:00 hours (8-9 am) if Bishop score is 6 or above. The induction involved artificial rupture of the fetal membranes with Kocher's forceps and synchronous oxytocin stimulation in escalating doses. The dose of oxytocin used for induction of labour was 10 IU in a 1000 ml bottle of normal saline. The infusion was started at 10 drops per minute (5 mIU per minute) using the British standard blood giving set. The dose was then titrated against the uterine contractions by increasing the rate by 10 drops every 30 minutes for patients who are para 0 to 4 and every 45 minutes for grand multiparous patients. Titration was continued until uterine contractions are established at a frequency of 3 in 10 minutes, or the maximum rate of 60 drops per minute (30 mIU per minute) was reached. Oxytocin titration was further increased if cervicogram touches the action line in escalating doses to achieve 5 contractions in 10 minutes to a maximum of 60 drops per minute. The oxytocin dose was reduced whenever 6 or more contractions in 10 minutes or contractions lasting 60 seconds or more occurred. If contractions were inadequate with 10 units of oxytocin, the dose was increased to 20 units in 1000 ml of normal saline and oxytocin titration reduced to 30 drops/minute and similarly used. A unit-modified partograph with the alert line drawn parallel from zero cervical dilatation and the action line drawn 4 hours to the right and parallel to the alert line was used to monitor the progress of induced labour. Intermittent fetal monitoring with Doppler fetal monitors (Sonicaid, Scotland)/fetoscope was used for fetal monitoring. Successful induction of labour was defined as a parturient achieving vaginal delivery within 16 hours of induction. Duration of labour was defined as the time interval between the attainment of active phase labour (from 4 cm cervical dilatation) and delivery of the fetus.

### 2.7. Sample Size (*n*)

The sample size was calculated using Cochran's formula [25].

(1) $$\begin{array}{c} n=Z^{2}p\frac{\left(1-p\right)}{e^{2}}, \end{array}$$

where *n* = sample size. *Z* = 1.95 at 95% level of confidence. *p* = estimated proportion = (2.9% = 0.029) [26]. *e* = level of precision (5% = 0.05). *n* = 1.96^2^ × 0.029 × (1 − 0.029)/0.05 × 0.05. *n* = 3.8416 × 0 .029 × 0.971/0.0025. *n* = 0.1081756/0.0025. *n* = 43.27 = 43. Attrition rate of 20% = 8.6. New sample size = 52; a sample size of 60 was adjudged to be adequate.

### 2.8. Data Analysis

The data were analyzed using the IBM SPSS Statistics 20 (IBM Corp., Armonk, NY, USA). Continuous variables were analyzed using simple percentages and student's *t*-test while categorical variables were represented using simple percentage and graph. The degree of association between preinduction cervical length and Bishop score with the induction delivery interval and mode of delivery were reported using chi-square (*X*^*2*^) and relative risk. These were done at *P* < 0.05.

## 3. Results

A total of 60 pregnant women who consented to the study were recruited, and during this period, the total number of deliveries was 1333 given an induction delivery rate of 4.5%. Induction of labour was successful in 100% of women recruited in the study. From Table 1, the demographic characteristics of the study population showed that the mean maternal age was 30.68 ± 6.38 years with a range of 19–43 years. The mean gestational age was 39.57 ± 1.49. Majority of the study population belongs to the age bracket of 20–29 years with a percentage of 41.7%. The mean parity in the study was 1.85 ± 0.63. Primigravida constituted 28.3% of the women while 13.3% of the study population was grand multiparous. The highest number of women (58.3%) had one to four (1–4) deliveries. Eighty-five, ninety-five, and more than sixty percent of the study population were Igbos, Christians, and urban dwellers, respectively.

**Figure 1:**
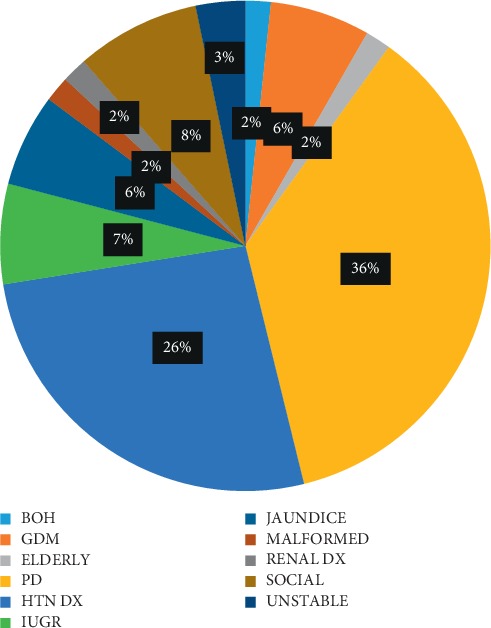
Indication for induction of labour. Key : BOH, bad obstetric history; GDM, gestational diabetes mellitus; ELDERLY, elderly primigravida; HTN DX, hypertensive disease in pregnancy; IUGR, intrauterine growth restriction; JAUNDICE, jaundice in pregnancy; MALFORMED, congenital malformation; PD, postdated pregnancy; RENAL DX, renal disease; SOCIAL, social induction of labour; UNSTABLE, unstable lie.

From Figure 1, twenty-two (22) parturients were induced on account of prolonged pregnancy with a percentage rate of 36.7%. The second highest indication was hypertensive disease complicating pregnancy with a percentage rate of 26.7%. 1.7% each had induction of labour on account of bad obstetric history, fetal malformation (anencephaly), and renal disease. Stabilization induction of labour was done for two women (3.3%) with five (5) women (8.3%) induced on account of social reasons. GDM and IUGR had a frequency rate of 4 (6.7%) and 4 (6.7%), respectively.

From Table 2, the mean preinduction cervical length, Bishop score, induction delivery time, and duration of labour were 2.9 ± 0.6 cm, 7.5 ± 1.0, 8.13 ± 3.0 hours, and 7.43 ± 2.9 hours, respectively. Fifty-two percent (51.7%) of the study population had Bishop score of seven (7) or below. The number of the study population with cervical length greater than three (>3) was 51.7% while 48.3% had a cervical length that was less than three (≤3). The mean duration of labour was 7.4 ± 2.9 hours with more than half (53.3%) of the women having a duration of labour lasting less than 6 hours. In more than two-fifth (46.7) of the women, the duration of labour lasted six or more hours. The mean induction delivery time was 8.1 ± 3.0 hours for the study population with 35 women (58.3%) delivering less than 8 hours following induction of labour. More than seventy-eight percent of the neonates had normal Apgar score while 3.3% had perinatal death. The mean neonatal weight was 2.18 ± 0.39 kg with the majority (81.7%) of the neonates weighing 2.5–3.5 kg.

**Figure 2:**
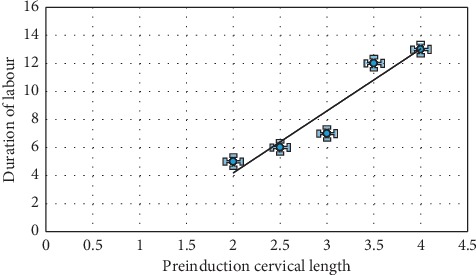
Association between induction delivery interval and sonographically measured cervical length.

Figure 2 above, induction delivery interval increases as the production cervical length increases. Preinduction cervical length of less than 3 cm is associated with a decrease in induction delivery time.

**Figure 3:**
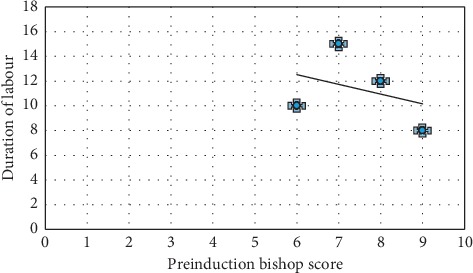
Association between induction delivery interval and clinically measured Bishop score.

As shown in Figure 3, as the Bishop score increases, the induction delivery time decreases.

From Tables 3 and 4, an equal variance *t*-test showed a statistically reliable difference between preinduction cervical length for duration of labour (<6 hours: *M* = 2.51, *S* = 0.49, *t* (58) = 3.207, *P* = 0.002, and ∞ = 0.05). However, an unequal variance independence test failed to show any statistical difference for preinduction Bishop score and duration of labour (≥6 hours: *M* = 7.48, *S* = 1.04; <6 hours: *M* = 7.56, *S* = 0.98, *t* (58) = 0.281, *P* = 0.780, ∞ = 0.05). Pearson correlation showed a positive correlation between preinduction cervical length and duration of labour (*R* = 0.681, *P* = 0.001, ∞ = 0.01) unlike preinduction Bishop score, which had a negative correlation (*R* = −0.159, *P* = 0.113, ∞ = 0.01). The positive predictive value of preinduction cervical length predicting a labour lasting less than six hours was 75%, negative predictive value of 82% with a sensitivity of 83%. Bishop score had a positive predictive value of 56%, negative predictive value of 53%, and a sensitivity of 58%.

As shown in the regression table (Table 5), the regression analysis table showed that preinduction cervical length was a significant explanatory variable to predict the response on duration of labour (*R* = 0.681, *R*^2^ = 46.4% *t* = 7.080, *P* = 0.001, ∞ = 0.05), although it could only account for 46.4% change in the dependent variable (duration of labour). The model fit for preinduction Bishop score showed a low correlation for the duration of labour (*R* = 0.012, *R*^2^ = 0.000, *t* = 0.088, *P* = 0.930, ∞ = 0.05). Layer by layer analysis with some of the confounding factors such as parity and maternal age on preinduction cervical length showed that grand multiparous women and women older than 40 years had increased regression coefficient. There was an increased percentage change in the duration of labour for each change in the predictor variable. This percentage change was most marked within the parity and age group of 1–4 (74.4%) and 20–29 (77.7%), respectively. These *R*^2^ values were larger than the baseline *R*^2^ value of 46.4% implying that the effect of cervical length was more marked in these group of the cohort of women studied.

## 4. Discussion

The commonest indication for induction of labour in this study was prolonged pregnancy, which is in keeping with other reports in Nigeria [27]. Most of the women that underwent induction in our study were at a gestational age greater than 39 weeks. This is in line with the current concept about “Term,” which was initially thought to be homogeneous in terms of fetal outcome, but a study done in America has refuted this thinking [28] showing that baby delivered in early term have increased morbidity compared to a full-term baby. It is now recommended that elective induction of labour should only be carried out at the gestational age of 39 weeks and above to reduce neonatal morbidity and mortality [28]. The average gestational age at the induction of 39 weeks and above in this study is not in keeping with the report by WHO global survey on maternal and neonatal health that reported that most of the induction of labour in Africa was done at a gestational age less than 39 weeks [29].

A study done in Enugu Southeast of Nigeria reported an increasing trend of induction of labour carried on maternal request [30]. The trend is evidenced in our study that showed one in twelve (1 : 12) women being induced on account of self-request. They belonged mostly to higher social class in agreement with a similar finding in Enugu, Southeast Nigeria [30]. This could be attributed to increasing awareness/education of the client and the willingness of the obstetrician to oblige their request. This calls for a proper appraisal because an alternative to induction of labour is a caesarean section. It should not be carried out on trivial reasons. The risk is very much especially in our region where the aversion to caesarean section is high with its obstetric or nonobstetric complications.

The incidence of induction of labour in our study was 4.5%, which fell within the range of incidence of labour in our environment [31] and agree on the incidence rate reported in Africa [32]. This ranged from 1.4% in Niger to 6.8% in Algeria unlike high incidence rates in Asia and the developed world [32]. This low incidence rate in Africa has been attributed to the high unmet need of about 66–80.2% for labour induction [29]. This has been attributed to a low threshold for caesarean section and lack of manpower, appropriate medication, lack of training of health worker, and lack of appropriate instrument to monitor a woman undergoing induction labour [32].

Traditionally, Bishop score is the method of choice in the assessment of cervical ripeness before the artificial induction of labour. It is however accepted that it has some limitations necessitating the thinking that ultrasound assessment of cervical findings could help limit its shortfalls [8]. This study has assessed the usefulness of preinduction cervical length versus Bishop score in the prediction of successful labour induction. Both methods of assessment in our study are associated with the successful induction of labour, which agrees with the work of Cubal et al. [33]. As might be expected, the duration of labour is markedly shorter with an increase in Bishop score. Bishop score is an expression of a parturient nearness to spontaneous onset of labour with increasing BS denoting closeness to active phase labour following labour induction. Even though our study showed no significant association between Bishop score and duration of labour (*x*^*2*^ = 0.577, df (1); *P* value = 0.448), the cohort of women with BS greater than 7 has 1.21% chances of delivering within 6 hours of commencement of IOL (RR = 1.2195% CI 0.734–2.001). On the other hand, a significant association was seen between CL and duration of labour with a higher significant linear correlation (*X*^*2*^ = 19.526; df (1) *P*=0.001; *R* = 0.681; *P* value = 0.001) than the findings with Bishop score. Parturient with cervical length ≤3 has a higher relative risk of short labour duration when compared with a longer CL. This finding in this study is supported by the work of Tan et al., which reported that ultrasound measured CL prediction has superior sensitivity in the prediction of successful IOL [11]. Support for this finding is also seen in other studies done in India [12], Turkey [13], Iran [14], Netherlands [8], and London [15]. Ben-Harush et al. [16] in a prospective study of 71 patients observed a statistically significant linear correlation as in our study between sonographic cervical lengths prior to IOL and labour duration. The cohort of women with CL of less than 28 mm significantly had a shorter induction delivery time compared to patients with more than 28 mm length. In a similar study although with a different route of CL measurement, Khazardoost et al. [17] also showed that higher cervical length is significantly associated with a parturient being delivered abdominally.

In addition to the above, Pandis et al. [34] demonstrated that cervical length performed better than Bishop score in the prediction of vaginal delivery. They reported that even though both parameters successfully predicted vaginal delivery, the positive predictive value of ultrasound measured cervical length is 20% higher than that of Bishop score. Parturient with cervical length less than 19 mm are less likely to remain undelivered than parturient with long cervical length. In terms of Bishop score, the only significant contributor is the effacement, which is also an index of cervical length. This is consistent with the original work of Bishop in 1964 that showed that a score of 9 or more in multiparous women is associated with a high chance of successful delivery [35]. Similar finding as in Pandis et al. [34] was noted in our study. In this study, the positive predictive value of preinduction cervical length predicting labour lasting less than six hours was 75%, which was 19% higher than the positive predictive value of Bishop score. In the study of Gabriel et al. [36], they reported that the use of cervical length measurement is more important in women with unfavourable Bishop score. Their study showed that cervical length is predictive of the mode of delivery in a parturient with Bishop score ≤5 but not in women with favourable Bishop score. This finding could be explained by the dynamic changes that occur in the cervix before the onset of labour. Cervical shortening concerns primarily at the supravaginal segment of the cervix, which is not accessible by digital vaginal examination especially when the cervix is closed. Preinduction sonographic assessment of this segment of the cervix (supravaginal) becomes more important in women with unfavourable Bishop score who seems to have undergone little cervical changes [23]. Bortha et al. [23] argued that short cervical length of less than 30 mm is a better predictor of cervical ripeness and therefore less need for cervical ripening. They reported that using Bishop score to traditionally define unripeness of the cervix leads to unnecessary use of prostaglandin to ripen the cervix. This is especially important in triaging for delivery when the indication for induction is “minor” or when there is urgent need to deliver the client with unfavourable Bishop score.

Our study was however not supported by the studies of Chandra et al. [18], Gonen et al. [19], Watson et al. [20], and Rozenberg et al. [21]; these studies showed no correlation between sonographic cervical length and induction delivery interval, duration of labour, successful induction, or length of the latent phase of labour. The possible reasons that could account for this difference with our study might be attributed to the heterogeneous population studied (population bias), study design, and difference in study endpoint. The above studies unlike in our study allow women with low Bishop score to be induced, even though they all noted the low predictive value of low Bishop score in predicting successful induction of labour. The heterogeneous population involved in these studies is compounded by the study design. The methods of induction used were not uniform, varying from amniotomy, oxytocin, and prostaglandin use unlike in our study where oxytocin titration in escalating doses with synchronous amniotomy was employed. All the cohort of women studied in our study had a successful vaginal delivery. The outcome might be attributed to the effect of patient selection or the strong desire to avoid caesarean section inherent in our area. In our study, parturient with cervical length >30 mm had long induction to delivery interval compared to a cohort of women with cervical length ≤30 mm. Ware and Raynor [37] found out that women with cervical length >30 mm were associated with an increased caesarean section rate with their labour lasting longer than those with low cervical length. Further analysis by Ware and Raynor [37] study showed that only 2% of women with cervical length less than 18 mm remaining undelivered following induction compared to 9% of women with Bishop score of 5–9.

Our study showed that 48.3% of women have a cervical length less than 3 cm while 51.7% has a cervical length greater than 3 cm; an independent test of equal variance shows a significant difference between these groups with induction delivery interval/duration of labour (*P* = 0.001). There is also a positive correlation between induction delivery interval/duration of labour and cervical length (*P* = 0.001). Bivariate analysis using Bishop score and cervical length as independent variables fail to show any significant association between Bishop score and induction delivery interval/duration of labour. The regression is low with 0% change on the independent variable for a unit change in the Bishop score. Some of the findings in this study agree with the study of Pandis et al. [34] and Gonen et al. [19] that reported that cervical length is a significant predictor of induction delivery interval. However, it differs with their findings of a significant association between Bishop score and successful induction of labour, although the finding is in keeping with the work of Boozarjomehri et al. [38]. Our study showed that a cervical length of less than 3 cm is associated with an increased relative risk for short induction delivery interval/duration of labour. This agrees with the report that successful induction of labour is associated with a short cervical length, which is a marker of cervical effacement [39]. It is the most important component of Bishop score in the prediction of successful induction of labour. The model fit analysis showed that although there is a positive functional relationship between preinduction cervical length and induction delivery interval, this could only account for less than fifty percent (<50%) change in the dependent variable for a unit change in the predictor variable. It could be deduced that other factors other than cervical length are involved in the prediction of induction delivery interval. This study also showed that increasing parity is a significant prediction of induction delivery interval.

## 5. Conclusion

The study has demonstrated that the use of transvaginal ultrasound is a good predictor of successful induction of labour than Bishop score. The study also shows that women with cervical length ≤30 mm are likely to have a short duration of labour following induction. This is important in triaging for induction of labour when the indication is “minor,” Bishop score not favourable, or there is an urgent need to induce labour. It could also assist the obstetrician in the counselling of the client on the prognosis of induction of labour.

## Figures and Tables

**Table 1:** Obstetrics and demographic characteristics of the study population (*n* = 60).

Characteristics	Frequency (%)
*Parity*	
0	17 (28.3)
1–4	35 (58.3)
>4	8 (13.3)

*Education*	
Primary	20 (33.3)
Secondary	19 (31.7)
Tertiary	21 (35.0)

*Residence*	
Urban	37 (61.7)
Rural	23 (38.3)

*Occupation*	
Government employed	18 (30.0)
Trading	15 (25.0)
None	15 (25.0)
Private employed	6 (6.0)
Self-employed	6 (6.0)

	Mean (range)
Maternal age (years)	30.6 ± 6.4 (19–43)
Gestational age (weeks)	39.5 ± 1.5 (36–42)

**Table 2:** Clinical/ultrasound findings.

Parameters	*n* (%)	Mean (SD)	Mode	Median
Bishop score		7.5 (1.02)	7.0	7.0
6-7	31 (51.7)			
>7	29 (48.3)			
Preinduction cervical length		2.9 (0.69)	2.03	3.01
<3 cm	29 (48.3)			
≥3 cm	31 (51.7)			

**Table 3:** Influence of the Bishop score on induction delivery interval.

Variable	Bishop score		*P* value
	≤7	>7	
Duration of labour			
<6 hours	18	14	0.465
≥6 hours	13	15	

*X* ^*2*^ = 0.577, df (1); *P* value = 0.448; *R* = 0.012 *P* value = 0.465. RR for cohort BS ≤ 7: 1.21(95% CI 0.734–2.001). RR for cohort BS > 7: 0.82(95% CI 0.484–1.377).

**Table 4:** Influence of the cervical length on induction delivery interval.

Variable	Cervical length		*P* value
	≤3 cm	>3 cm	
Duration of labour			
<6 cm	24	8	0.001
≥6 cm	5	23	

*X* ^2^ = 19.526; df (1) *P* = 0.00001; *R* = 0.681 *P* value = 0.001. RR for cohort CL ≤ 3: 4.20 (95% CI 1.85–9.529). RR for cohort CL > 3: 0.30(95% CI0.163–0.568).

**Table 5:** Regression analysis of preinduction cervical length vs. duration of labour with confounding variables.

Statistics	Nil	Parity			Age			
		0	1–4	>4	<20	20–29	30–39	≥40
*Model fit*								
Regression	0.681	0.701	0.639	0.794	1.000	0.552	0.745	0.849
*R* ^2^ (%)	46.4	67.5	74.4	65.5	67.4	77.7	55.4	72.1
Std error estimate	2.13	2.22	2.312	1.239	—	2.303	2.049	1.583

*ANOVA*								
*F* value	50.128	14.505	22.732	10.209	—	10.07	27.377	18.092
*P* value	0.001	0.002	0.001	0.019	—	0.004	0.001	0.004

*Coefficient*								
*t*-value	7.08	3.809	4.768	3.195	—	3.174	5.232	4.253
*P* value	0.001	0.002	0.001	0.019	—	0.004	0.001	0.004

## Data Availability

All data generated or analyzed during this study are included in this published article.

## Additional Points

One of the limitations of this study is its cross-sectional nature and the small sample size, which limit the conclusion that one can draw from the study. Increasing the sample is advocated in the study area to help authenticate our findings. Because it is a hospital-based study, the finding of this study could not be generalized in the area of study; this could be circumvented by conducting further research on this subject involving recruitment of participant from other mission hospitals in the area and even conducting a randomized controlled study on the place of cervical length on the induction of labour.
